# Highly Water-Soluble Solid Dispersions of Honokiol: Preparation, Solubility, and Bioavailability Studies and Anti-Tumor Activity Evaluation

**DOI:** 10.3390/pharmaceutics11110573

**Published:** 2019-11-01

**Authors:** Li Wang, Weiwei Wu, Lingling Wang, Lu Wang, Xiuhua Zhao

**Affiliations:** 1 College of Chemistry, Chemical Engineering and Resource Utilization, Northeast Forestry University, Harbin 150040, Heilongjiang, China; kobe4813765@163.com (L.W.); wuweiwei0522@163.com (W.W.); llwang@nefu.edu.cn (L.W.); tdcq0707@163.com (L.W.); 2 Key Laboratory of Forest Plant Ecology, Northeast Forestry University, Ministry of Education, Harbin 150040, Heihongjiang, China

**Keywords:** honokiol, solid dispersion, solubility, bioavailability, antitumor activity

## Abstract

Honokiol (HK), a well-tolerated natural product, has many multiple pharmacological activities. However, its poor water solubility and low bioavailability limit its clinical application and development. The aim of this research was to prepare the solid dispersion (SD) formulation of honokiol (HK) with poloxamer-188 (PLX) as the carrier, thereby improving its solubility and oral bioavailability. Firstly, by investigating the relationship between the addition amount of the PLX and the solubility of HK, and the effects of solid dispersions with different ratios of HK–PLX on the solubility of HK, we determined that the optimum ratio of PLX to HK was (1:4). Then, the HK–PLX (1:4) SD of HK was prepared using the solvent evaporation method. The morphology of the obtained HK–PLX (1:4) SD was different from that of free HK. The HK in the HK–PLX (1:4) SD existed in amorphous form and formed intermolecular hydrogen bonds with PLX. Additionally, the solubility values of the HK–PLX (1:4) SD were about 32.43 ± 0.36 mg/mL and 34.41 ± 0.38 mg/mL in artificial gastric juice (AGJ) and in artificial intestinal juice (AIJ), respectively. Compared with free HK, the release rate and the bioavailability was also substantially improved for HK in its SD form. 3-(4,5-dimethylthiazol-2-yl)-2,5-diphenyltetrazolium bromide (MTT) assay indicated that the HK–PLX (1:4) SD showed higher inhibition of HepG2 cells than free HK. Taken together, the present study suggests that the HK–PLX (1:4) SD could become a new oral drug formulation with high bioavailability and could produce a better response for clinical applications of HK.

## 1. Introduction

Today, in the process of drug development, low water solubility is still a difficult problem that needs to be solved for drug candidates. Many insoluble drugs are lipid-soluble drugs with low water solubility and high permeability [1]. So far, various methods have been explored to enhance the dissolution properties of these insoluble drugs, such as using salt formation [2], cyclodextrins [3], self-emulsifying drug delivery systems (SEDDS) [4], liposomes [5], micelles [6], co-crystals [7], micro- or nanoparticles [8], and solid dispersion [9]. Among them, solid dispersion is a relatively more sophisticated technology that overcome the limitations of insoluble drugs in FDA available products. The solid dispersion technology recently reported in the literature and used in approved products is also becoming more popular [1]. Solid dispersion formulations with hydrophilic excipients have many merits, such as enhanced wettability, higher porosity, and having an amorphous state [10].

**Figure 1:**
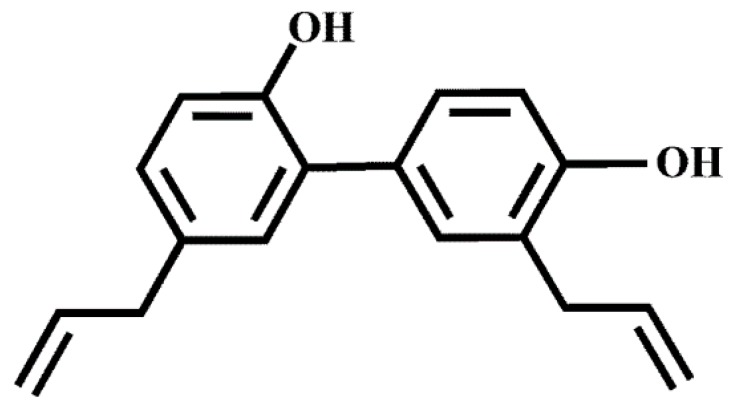
Chemical structure of honokiol.

Generally, the formation of intramolecular hydrogen bonds in the drug molecules reduces the polarity of the molecules, thus reducing the solubility in water. The formation of intermolecular hydrogen bonds increases the solubility of the drugs, probably because the formation of hydrogen bonds between drug molecules and water molecules accelerates the dissolution of the drugs. Honokiol (HK) is a bioactive natural compound extracted from the stem, bark, and roots of *Magnolia officinalis* [11]. It is a small biphenolic lignan (Figure 1) with the molecular formula C_18_H_18_O_2_ [12]. HK exists in nature as a white crystalline powder, and its melting point is about 85 °C [13]. Moreover, HK has many diverse pharmacological and biological properties, including anti-anxiety effects, anti-oxidant actions, anti-inflammatory effects, and anti-cancer effects [14,15,16]. HK has been extensively researched and developed because of its numerous applications and good safety. However, the phenolic hydroxyl groups of HK are prone to forming intramolecular hydrogen bonds, which lead to decreased solubility in water, thus limiting its application for various diseases [17,18]. If a carrier-containing oxygen or hydroxyl group forms intermolecular hydrogen bonds with HK, then the hydrogen bonds between the drugs and the carriers hinder the formation of intermolecular interaction between drug molecules, and as such the solubility of HK is improved. 

Hydrophilic poloxamer-188 (PLX) is a polyoxyethylene–polyoxypropylene copolymer. Due to its low melting point, high drug loading, good hydrophilicity, and good safety, PLX is not only widely used in some traditional formulations, such as suppositories, tablets, and emulsions, but also can form micelles to increase the solubility of insoluble drugs, increase the stability of drugs, and promote the absorption of drugs by the body. Additionally, it is a surfactant approved by the FDA [19]. Moreover, PLX is also a commonly used carrier material for poorly water-soluble PLX is also a commonly used carrier material for poorly water-soluble drugs, which can ensure the high dispersibility of drugs in water and prevent drug aggregation [20,21]. Compared with polyvinylpyrrolidone (PVP) K30, polyethylene glycol (PEG) 6000/4000, and Soluplus, the viscosity of PLX is lower, which means the solution is easily desiccated. Moreover, for some poorly water-soluble drugs, the solid dispersion of PLX as a carrier has a higher solubility than other polymers, such as PEG 4000, PEG 6000, and PVP K30 [10,22]. Considering these properties, PLX can be recommended as a potential hydrophilic carrier for solid dispersion. 

In addition, some technologies have been used to enhance the solubility and oral bioavailability of free HK. For example, Xu and coworkers prepared an inclusion complex of HK with sulfobutyl ether-β-cyclodextrin (SB-β-CD), and in vivo results showed that the AUC(0–t) and *C*_max_ of the inclusion complex increased by approximately 158% and 123%, respectively, compared with free HK [14]. Han and coworkers prepared honokiol nanosuspensions using a solvent precipitation–ultrasonication method, and the nanosuspensions improved the oral bioavailability of honokiol in in vivo studies in rats, showing a 3.94-fold *C*_max_ increase and a 2.2-fold AUC (0–t) increase [15]. Godugu and coworkers prepared honokiol-loaded nanomicelles (HNK-NM) using amphiphilic polymer Vitamin E Polyethylene Glycol Succinate. The nanomicellar formulations resulted in a significant increase in the oral bioavailability. *C*_max_ increased by 4.06- and 3.60-fold and AUC by 6.26- and 5.83-fold in comparison to 40 and 80 mg/kg oral application of free HK, respectively [23]. In addition, Zhang and coworkers prepared the pectin nanoparticles loaded with honokiol and the in vitro release results indicated that the drug-loaded nanoparticles exhibited a higher drug release rate than free honokiol, showing effective sustained release [24]. Nevertheless, following these strategies, there is still a need for continuous exploration in terms of dissolution and solubility enhancement, and further studies are required for new formulations and to explore the development process. Solid dispersion (SD) technology is an effective and economical method to improve the solubility and release properties of drugs, and its preparation process is easy, simple, and reproducible. Hence, it is feasible to develop a new HK SD system with good water solubility, low toxicity, and which is suitable for clinical application and mass production. Moreover, the preparation of an oral SD formulation of HK using PLX as the carrier and the effects of this formulation on the physicochemical properties, solubility, and oral bioavailability of the HK have not been studied so far.

In recent years, various technologies, such as melting method [25], spray-drying method [26], supercritical fluid method [27], hot melt extrusion technologies [28], solvent evaporation method [29,30], and freeze-drying method [31,32], have been extensively used in the preparation of SDs. However, some of these approaches have many disadvantages. For example, the melting method, spray-drying method, and hot melt extrusion method require high preparation temperature, which can cause degradation of the drugs. Further, the entire amount of drug used in the preparation cannot be completely melted into the carrier materials. In addition, the freeze-drying method and the supercritical fluid method require large equipment and high cost investment. Compared with other methods, the solvent evaporation approach has the advantages of low cost and convenient operation and regeneration, and has the potential to realize industrial production.

In the present investigation, a HK–PLX solid dispersion was prepared using solvent evaporation method, and its physicochemical properties were characterized by SEM, FTIR, XRD, and DSC. Furthermore, the dissolution in vitro, the solubility and the bioavailability in vivo, and the MTT assay were also investigated and evaluated. 

## 2. Materials and Methods 

### 2.1. Materials 

Poloxamer-188 (PLX), hydroxypropyl methylcellulose (HPMC), polyethylene glycol (PEG) 4000, PEG 6000, and polyvinylpyrrolidone (PVP) K30 were purchased from Aladdin (Shanghai, China). Honokiol (purity = 98.5%) was obtained from Baoji Haoxiang Biotechnology Co., Ltd (Baoji, China). Ethanol, acetonitrile, and methanol were obtained from Shandong Yuwang Industrial Co., Ltd (Shandong, China).

### 2.2. Preparation of the HK SD Formulation

Initially, the HK and the PLX were dissolved in ethanol for 5 min to reach the clear organic phase, and then the solution was stirred for 30 min under ambient conditions. After 30 min, the ethanol was evaporated using a rotary evaporator (SENCO, Shanghai, China) at 35 °C to obtain the SD formulation of HK with PLX (HK–PLX SD), and the obtained HK–PLX SD was then dried in a vacuum oven to remove the residual solvent. The removed ethanol could be recycled and reused. Finally, the SDs with different mass ratios of HK/PLX were obtained through the same process. The short names for as-synthesized HK SDs are as follows: HK–PLX 2:1, HK–PLX 1:1, HK–PLX 1:2, HK–PLX 1:3, HK–PLX 1:4, HK–PLX 1:5, HK–PLX 1:6, and HK–PLX 1:7. 

In addition, the SD formulation of the HK and other carriers (the mass ratio of HK to carriers was 1:5), including HPMC, PEG 4000, PEG 6000, and PVP K30, were also prepared via solvent evaporation method.

### 2.3. Characterization of the HK SD Formulations

#### 2.3.1. Scanning Electron Microscopy (SEM)

Apparent morphology of the HK–PLX (1:4) SD and the free HK was examined by a SEM (FEI, Eindhoven, Netherlands). Dry samples were fixed on the sample tables with a conductive paste and made to be electrically conductive by sputter-coating with gold using the ion sputtering coating machine. 

#### 2.3.2. Fourier Transform Infrared Spectroscopy (FTIR)

The structure of the HK–PLX (1:4) SD, the physical mixture (PM), and the free HK were examined in the scanning range of 400–4000 cm^−1^ by FTIR spectrophotometer (SHIMADZU, Kyoto, Japan). Each sample (2 mg) was accurately weighed and mixed with KBr (200 mg) and then pellets were made to perform the measurements. 

#### 2.3.3. X-Ray Diffractometry (XRD)

The crystal forms of the HK–PLX (1:4) SD, the PM, and the free HK were tested and evaluated by an X-ray diffractometer (Rigaku Corporation, Tokyo, Japan). Each sample was measured in the range of 5–60°. Appropriate process parameters were: scanning speed 5°/min; generator current 30 mA; generator tension (voltage) 40 kV. 

#### 2.3.4. Differential Scanning Calorimetry (DSC). 

Thermal characteristics of the HK–PLX (1:4) SD, the PM, and the free HK were detected by DSC (TA instruments, New Castle DE, USA). Samples of 4–6 mg were placed in a sealed aluminum pan and then heated from 40 °C to 400 °C in nitrogen atmosphere at a heating rate of 10 °C/min. 

#### 2.3.5. Gas Chromatography (GC) Measurement

The solvent residue of ethanol in the HK SD was inspected by an Agilent 7890A gas chromatograph (Agilent Technologies, Palo Alto, CA, USA). Firstly, 100 mg of HK SD powder was weighed and dissolved in acetone (1 mL), and then centrifuged at 10,000 rpm for 10 min to obtain the supernatant. Then, 5 μL of the supernatant was injected into a GC system for analysis, with a split ratio of 20:1. The detection conditions were as follows: initial temperature was kept at 40 °C for 5 min, then the temperature was raised to 100 °C for 2 min at 10 °C/min, then 240 °C at 40 °C/min, and then maintained for 2 min. In addition, both the inlet and detector temperatures were set to 250 °C. The air flow rate was 400 mL/min, the nitrogen flow rate was 25 mL/min, and the hydrogen flow rate was 30 mL/min.

### 2.4. Solubility Tests of the HK SD Formulations

#### 2.4.1. High Performance Liquid Chromatograph (HPLC) Method for HK

HK was quantified by a Waters 1525-2489 high performance liquid chromatograph (Waters Corporation, Milford, MA, USA) consisting of a pump (Waters 1525 binary) and UV detector (Waters 2489 Tunable Absorbance Detector), which was equipped with a Diamonsil C_18_ reverse-phase column (4.6 × 250 mm, 5 μm, DIKMA, Beijing, China). A methanol (55%), acetonitrile (20%), and water (25%) mixture was used as mobile phase. Other parameters of HPLC detection were as follows: A wavelength was 294 nm, the flow rate of the mobile phase was 1 mL/min, and the injection volume was 10 μL.

A standard solution of HK was prepared as follows: HK (10 mg) was accurately weighed and dissolved in 10 mL methanol solution, and then the solution was diluted appropriately to obtain solutions of 0.5, 0.25, 0.125, 0.0625, 0.03125, 0.015625, 0.007813, and 0.003906 mg/mL. Using the concentration of HK standard solution as the abscissa and the absorbency as the y-coordinate, the linear chart was constructed, and the regression equation was *Y* = 11326308.72*X* − 8679 (*R*^2^ = 0.9998). The retention time of the HK was about 10–11 min.

#### 2.4.2. Solubility Tests

Solid dispersions of HK with different polymers (the mass ratio of HK to polymer was fixed at 1:5) were also prepared by solvent evaporation method. Each solid dispersion (the content of HK was excessive) was weighed and placed in small beakers with 2 mL of deionized water, and then the beakers were sealed and placed in a water bath at 37 ± 1.0 °C for 4 h with constant stirring (100 rpm). After 4 h, the samples were taken out and centrifuged at 12,000 rpm for 10 min, and then filtered through a 0.22 μm membrane filter. The supernatant obtained was used for HPLC to determine HK concentration.

The solubility of the SD of HK with PLX and the corresponding PMs in water were determined by HPLC detection. Firstly, the excess amounts of SD and PM samples (equivalent to 150 mg of free HK) were placed in 25 mL beakers, and then 10 mL distilled water was added to each beaker. Then, the beakers were sealed and placed in a 37 ± 1.0 °C water bath for 4 h with constant stirring (100 rpm). After 4 h, the samples were taken out and centrifuged at 12,000 rpm for 10 min, and then filtered through 0.22 μm membrane filter to remove the possible residual drugs. The final supernatant was assayed for HK by the HPLC system, as described above. 

### 2.5. Dissolution Tests

The equilibrium solubility studies of HK–PLX (1:4) SD, free HK, and the PM in artificial gastric juice (AGJ) (pH 1.2) were carried out and investigated with 0.4% Tween-80 and artificial intestinal juice (AIJ) (pH 6.8). Briefly, excess samples were weighed and placed in small vials containing 2 mL dissolution media. Then, each vial was placed in a 37 °C water bath and stirred for 48 h at 100 rpm. After 48 h, the samples were centrifuged at 12,000 rpm for 10 min and then filtered through 0.22 μm membrane filters. The final supernatant was diluted with methanol, and then the concentration of HK in the samples was determined by HPLC detection.

The in vitro release of the HK–PLX (1:4) SD, the PM, and the free HK was analyzed in the two media mentioned above. Dissolution experiments were consistent with the sink conditions and were carried out at 37.0 ± 0.5 °C at a rotation speed of 100 rpm. Samples equivalent to 200 mg of HK were weighed and dispersed in a 250 mL beaker containing 200 mL of dissolution media. One milliliter of sample was taken out at predetermined times of 5, 10, 15, 20, 30, 45, 60, 90, 120, 240, 480, and 720 min, and the corresponding volume of dissolution medium was supplemented. The samples obtained were centrifuged at 12,000 rpm for 10 min and then filtered through 0.22 μm membrane filters to separate the excess drug. The final supernatant was diluted with methanol, and then the concentration of HK in the samples was determined by the HPLC detection.

### 2.6. In Vivo Pharmacokinetic Study

#### 2.6.1. Animal Dosing

Eighteen Sprague–Dawley rats (200–220 g) were maintained at 25 ± 2 °C and 50–60% relative humidity (RH) under natural light/dark conditions for one week before the experiment. In the experiment, the rats were randomized into three different groups, administered orally with free HK, the HK–PLX (1:4) SD, or the PM (at the dose of 50 mg/kg). The experimental protocols were approved by the Institutional Animal Care and Use Committee of Harbin Medical University (approval No. HMUIRB-2008-06) on June 23, 2006.

Blood samples were collected from the retro-orbital plexus at different time intervals of 0.08, 0.17, 0.25, 0.33, 0.5, 1, 2, 3, 4, 6, 8, 12 and 24 h. Samples were collected in 1.5 mL centrifuge tubes containing heparin, and were then centrifuged at 3000 rpm for 10 min. The upper plasma was taken and stored in a refrigerator at −40 °C until analysis.

#### 2.6.2. Determination of HK Content in Plasma Samples

Plasma samples (200 μL) were placed in a clean 1.5 mL centrifugal tube. Then, 400 μL acetonitrile was added and vortexed for 30 s to precipitate plasma proteins, which was then centrifuged at 12,000 rpm for 10 min to separate the supernatant. Then, 50 mg sodium chloride was placed in a new centrifugal tube containing the supernatant. All the samples were placed in a 25 °C water bath for 10 min and then centrifuged at 12,000 rpm for 5 min. Finally, 10 μL of supernatant was analyzed for the amount of HK by HPLC method. 

### 2.7. MTT Assay

Inhibition rate of the HK–PLX (1:4) SD on HepG2 cell lines was evaluated by conventional methyl thiazole tetrazolium (MTT) cell survival assay. The assay was based on the reduction of MTT by the mitochon-drial dehydrogenase of viable cells into purple formazan crystals, which were dissolved in dimethyl sulfoxide (DMSO), and absorbance was then detected by the enzyme mark analyzer instrument. Firstly, HepG2 cell lines were seeded at a density of 1 × 10^4^ cells per well in 96-well plates and incubated for 24 h. Subsequently, the old medium was removed and the samples containing free HK and the HK–PLX (1:4) SD at different HK concentrations (HK: 900.0, 450.0, 225.0, 112.5, 56.25, 28.13, 14.06, and 7.03 μg/mL) were added to the plates (the samples containing free HK and the HK–PLX (1:4) SD with different HK concentration were dissolved with culture medium, and the obtained samples were filtered through a 0.45 μm pore diameter membrane, and then added in the plates). In addition, the corresponding PLX at different concentrations (PLX: 3600.0, 1800.0, 900.0, 450.0, 225.0, 112.52, 56.24, and 28.12 μg/mL) was also added to the plates. After 48 h incubation, the cells were washed and the fresh medium containing MTT was added into each plate, followed by incubation for another 4 h. Thereafter, the medium was removed and the formazan crystals were solubilized with DMSO (150 µL). After mild shaking for 10 min, the absorbance value (OD) was detected by the enzyme mark analyzer instrument (detection wavelength of 490 nm and reference wavelength of 630 nm) and compared with the blank control group. 

### 2.8. Statistical Analysis

The data were presented as mean ± standard deviation. The statistical analysis was analyzed by using one-sample *t*-test in the Origin software. A value of * *p* < 0.05 was accepted as statistical significance. For in vitro studies, 3–4 replicates were used each time. Three individual experiments were performed and results were averaged. For in vivo experiments, *n* = 6 was used.

## 3. Results and Discussion

### 3.1. Solubility Enhancement

#### 3.1.1. Solubility of Different Solid Dispersions of HK

**Figure 2:**
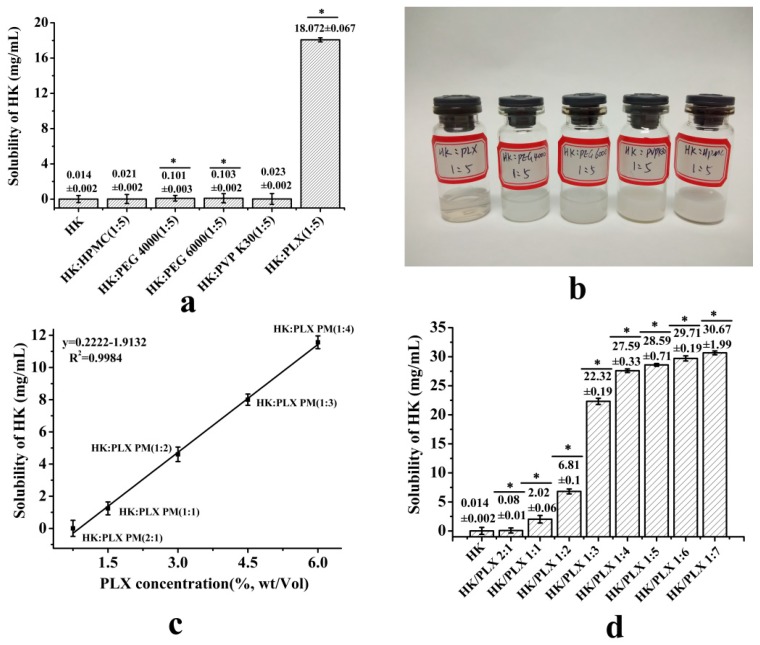
(**a**) The solubility of the different solid dispersions of honokiol (HK) (* *p* < 0.05). (**b**) Photographs of different solid dispersions in water. (**c**) Drug solubility as a function of poloxamer-188 (PLX) concentration in HK–PLX physical mixture. (**d**) The solubility of HK SD with different proportions of PLX (* *p* < 0.05).

In the experiments, we investigated the effects of different polymers, namely HPMC, PEG 4000, PEG 6000, PVP K30, and PLX, as carriers on the solubility of HK, and the measured solubility of the samples was shown in Figure 2a. As seen in the figure, the solubility values of the free HK and the different solid dispersions of HK were about 0.014 ± 0.002 mg/mL (free HK), 0.021 ± 0.002 mg/mL (HK–HPMC 1:5), 0.101 ± 0.003 mg/mL (HK–PEG 4000 1:5), 0.103 ± 0.002 mg/mL (HK–PEG 6000 1:5), 0.023 ± 0.002 mg/mL (HK–PVP K30 1:5), and 18.072 ± 0.067 mg/mL (HK–PLX 1:5). As you can see from these results, the solubility of solid dispersion with PLX as the carrier was significantly higher than that of other solid dispersions and free HK, and was 1290.86 times that of the free HK. The solubility values of other solid dispersions were a little higher than that of free HK (1.5–7.35 times higher than that of free HK).

In addition, the appearance of different solid dispersions (their quality was identical) in water is illustrated in Figure 2b. In the figure, the HK–PLX 1:5 was clearer and more transparent than other solid dispersions. Therefore, in this paper, the PLX was chosen as the carrier.

#### 3.1.2. Solubility of PMs and SD of HK

The measured solubility of free HK, PMs of HK with PLX, and HK SD are shown in Figure 2c,d. As seen in Figure 2d, free HK aqueous solubility was about 0.014 ± 0.002 mg/mL after 4 h at 37 °C. In addition, the critical micelle concentration (CMC) of the PLX was about 4.8 × 10^−4^ M [33]. As might be expected, the solubility of HK was linearly related to the concentration of surfactants above the CMC, which was in accordance with the micelle solubility equation *ST* = *S*0 + *k* (*P*_T_ − *CMC*) [34], as shown in Figure 2c. When the content of PLX was 6% *w*/*v*, the solubility of HK was about 11.56 ± 0.23 mg/mL, which was about 825.7 times that of free HK. It was expected that the solubility of HK would increase linearly with the further increase of PLX content, but that was not the case. When the content of PLX was 7.5%, 9%, and 10.5% *w*/*v*, the solubility of HK was about 12.4 ± 0.15, 11.9 ± 0.24, and 12.9 ± 0.12 mg/mL, respectively, and the magnitude of solubility enhancement was inconspicuous. Additionally, considering that the presence of large doses of surfactants in pharmaceutical products might cause unpredictable side effects in vivo, the amount of PLX did not need to be added too much.

Conversely, as expected, SD could enhance the solubility of HK more effectively. It was noteworthy that the solubility of the SDs in Figure 2d showed a significant increase; the difference between the different SDs was statistically significant (*p* < 0.05), and was obviously higher than that of the same proportion of PMs. As could be seen, the drugs existed in the SD in amorphous form, which was the critical factor for solubility enhancement. Additionally, when the mass ratio of HK to PLX was 1:4, the solubility of the SD was about 27.59 ± 0.33 mg/mL, while when the mass of PLX increased further, the solubility of the corresponding SD changed inconspicuously. Consequently, in this paper, the HK–PLX (1:4) SD was selected and used for the following detections. 

### 3.2. Characterization of HK Formulations

#### 3.2.1. SEM Results

**Figure 3:**
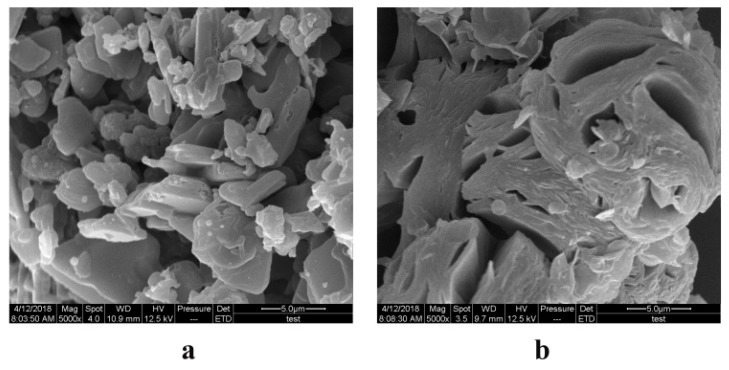
Scanning electron microscopy (SEM) images of the samples: (**a**) SEM image of free HK; (**b**) SEM image of the HK–PLX (1:4) SD.

The free HK and the HK–PLX (1:4) SD were detected by SEM to observe their morphology, as shown in Figure 3. The SEM image of free HK showed irregular block particles of various sizes (Figure 3a). However, the HK–PLX (1:4) SD showed block particles with porous structure (Figure 3b), and the morphology was different from that of free HK. Moreover, it could be seen from the figure that no HK crystals were observed in the solid dispersion, which suggested that the HK might be completely dispersed in the carrier material.

#### 3.2.2. Physicochemical Properties Characterization

**Figure 4:**
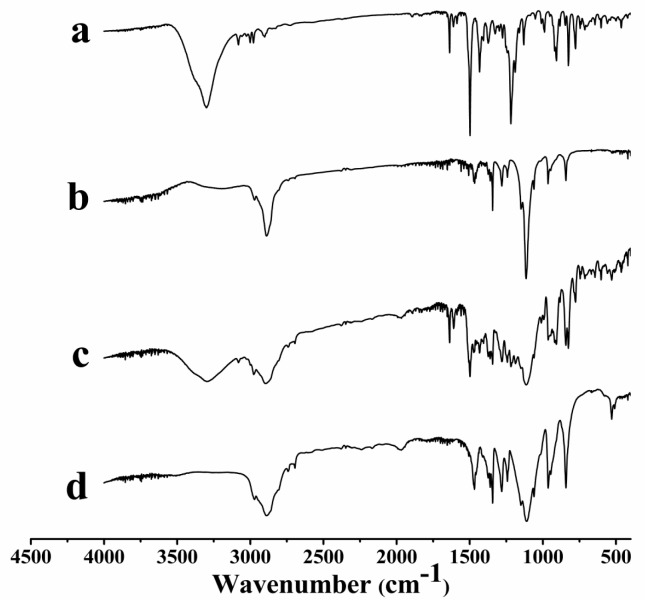
FTIR results (wavenumbers: 400–4000 cm^−1^) of the samples: (**a**) free HK; (**b**) HK–PLX (1:4) solid dispersion (SD); (**c**) physical mixture (PM) of HK and PLX; (**d**) PLX.

The possible interaction between HK and PLX was determined by FTIR. The FTIR spectra of free HK, the HK–PLX (1:4) SD, PM of HK with PLX, and PLX from 4000 to 400 cm^−1^ are shown in Figure 4. The free HK (curve a) exhibited characteristic peaks at 3304 cm^−1^ (–OH vibration), 1638 cm^−1^ (alkene C=C vibration), 1498 cm^−1^ (C=C aromatic stretching), 1217 and 908 cm^−1^ (C–O), and 989 and 825 cm^−1^ (C–C). The FTIR spectra of PLX (curve d) were characterized by principal absorption peaks at 2885 cm^−1^ (C–H stretch aliphatic), 1338 cm^−1^ (in-plane O–H bend), and 1106 cm^−1^ (C–O stretching). As for PM of HK with PLX (curve c), the spectrum basically consisted of the overlapping peaks of HK and PLX. Some characteristic absorption peaks of HK at 3303, 1638, 1497, 906, and 825 cm^−1^ were easy to find in PM, suggesting there was no interaction between HK and PLX. Meanwhile, these characteristic peaks of HK almost disappear in the spectrum of SD (curve b), which could be attributable to interaction between HK and PLX. Moreover, considering the chemical structures of PLX and HK, the hydrogen bonds may be formed between PLX and HK during the formulation of the HK–PLX (1:4) SD, and this interaction played a vital role in solubilizing the drug.

**Figure 5:**
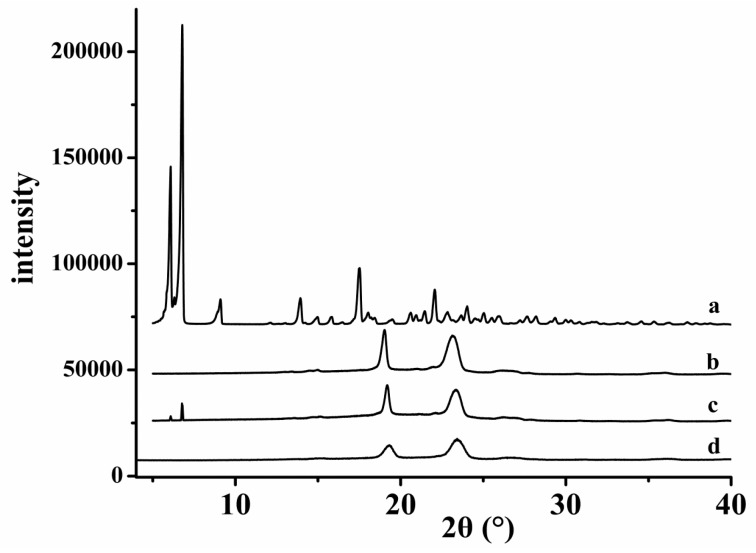
XRD results (2θ = 5–40 °) of the samples: (**a**) free HK; (**b**) HK–PLX (1:4) SD; (**c**) physical mixture of HK and PLX; (**d**) PLX.

**Figure 6:**
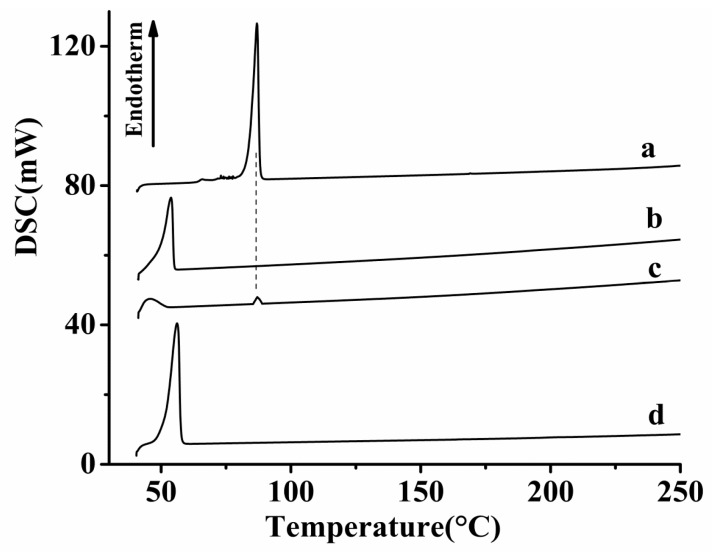
DSC results (temperature range: 30–250 °C) of the samples: (**a**) free HK; (**b**) HK–PLX (1:4) SD; (**c**) physical mixture of HK and PLX; (**d**) PLX.

The XRD and DSC results of the samples, including free HK, HK–PLX (1:4) SD, PM of HK with PLX, and PLX, are shown in Figure 5 and Figure 6, respectively. It can be seen in Figure 5a that free HK had many obvious diffraction peaks between 5° and 40°, and its DSC curve (Figure 6a) displayed one endothermic peak for the melting point at 86.7 °C, illustrating that the drug existed in nature as a crystal. Additionally, the diffraction patterns of PLX (Figure 5d) had two distinct diffraction peaks at 2*θ* = 19.23° and 23.4°, and as seen in the DSC curves of PLX in Figure 6d, the melting point peak of PLX was about 53.8 °C, indicating that PLX existed as a crystal. The XRD spectrum of the PM (Figure 5c) was basically superimposed with those of HK and PLX, and some diffraction peaks of HK still existed (considering the PLX dilution effect, the drug peaks almost disappeared due to the high percentage of PLX). Moreover, the DSC curve of the PM (Figure 6c) existed as two sharp endothermic peak at 53.8 and 86.9 °C, corresponding to the melting peaks of PLX and HK, indicating that HK existed in PM in the form of crystal. The endothermic peak of HK was relatively small, which was probably because the PLX first melted at 53.8 °C during the heating process of DSC. Some of the HK might have dissolved in the PLX, so the endothermic peak of HK in the PM was not obvious. As seen in Figure 5b, the endothermic peak of HK almost completely disappeared, and the endothermic peak shown in the spectrum of HK–PLX (1:4) SD was similar to that of PLX, indicating that HK might exist in the SD in an amorphous form, which was also consistent with XRD analysis.

In addition, the crystal structures of the SDs of HK prepared in different batches were also investigated, as shown in the supplementary materials (Figure S1). The results obtained showed that the HK could maintain its amorphous form in SD for a long time, which illustrated that the HK-PLX(1:4)-SD had good stability.

#### 3.2.3. Residual Solvent Determination

**Figure 7:**
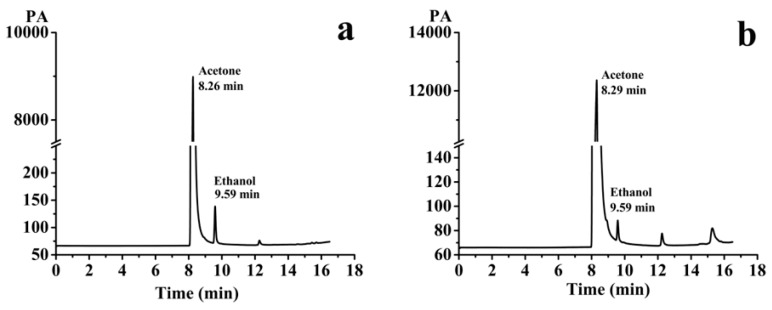
(**a**) Gas chromatograms of ethanol standard solution; (**b**) The gas phase of the 100 mg/mL acetone solution of HK–PLX (1:4) SD.

In this work, the ethanol with low toxicity was applied to prepare the HK–PLX (1:4) SD. Figure 7a,b display the results of the determination of residual ethanol solvents in the HK–PLX (1:4) SD by GC detection. In addition, a linear regression equation of peak area (Y) versus ethanol concentration (*x*) was obtained as Y = 1980.6 x + 221.13 (R^2^ = 0.999) over the range of 0.015625–1 mg/mL. According to the regression equation, the residual ethanol content in the HK–PLX (1:4) SD was 1733 ppm, which met the minimum standard (5000 ppm) required by the International Conference on Harmonization (ICH) and was suitable for pharmaceutical use.

### 3.3. Dissolution Study

**Figure 8:**
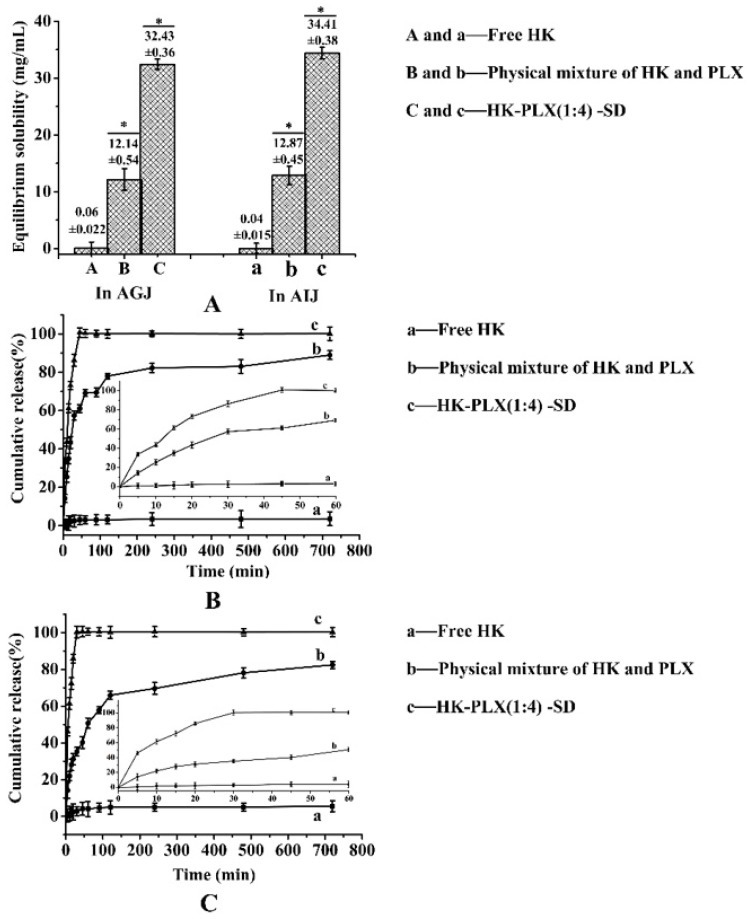
Equilibrium solubility (* *p* < 0.05) (**A**) and the release profiles in artificial gastric juice (**B**) and artificial intestinal juice (**C**) for free HK, the physical mixture, and the HK–PLX (1:4) SD.

The drug solubility and dissolution are displayed in Figure 8. As seen in Figure 8A, the equilibrium solubility values of the free HK, the PM of HK, and PLX in AGJ and AIJ were about 0.06 ± 0.022, 12.14 ± 0.54 mg/mL, 0.04 ± 0.015, and 12.87 ± 0.45 mg/mL, respectively. This illustrates that the PLX was beneficial in increasing the solubility of HK, which might be because PLX, as a surfactant, could increase the solubility of the drugs by forming micelles. However, the HK–PLX (1:4) SD values assessed by the HPLC detection of about 32.43 ± 0.36 mg/mL and 34.41 ± 0.38 mg/mL in AGJ and AIJ, respectively, were obviously higher than the others, and the difference between the groups was statistically significant (*p* < 0.05), indicating that the HK existed as an amorphism in HK–PLX (1:4) SD and formed intermolecular hydrogen bonds with PLX. So, the HK–PLX (1:4) SD had better dissolution and bioavailability.

Furthermore, the SD formulation of HK significantly improved drug dissolution compared to the drug powder. The release profiles of HK from the SD formulation compared to free HK are shown in Figure 8B,C. It was found that the release percentage of HK was extremely low in AGJ (pH 1.2) and AIJ (pH 6.8) and the maximal percentages of the drug released at 12 h were 3.57% ± 0.36% and 5.48% ± 0.25%, respectively. The PM of HK and PLX was dissolved 88.96% ± 0.28% and 82.41% ± 0.43% in AGJ and AIJ at 12 h, and the dissolution rate of the PM was faster than that of free HK, indicating that the PLX could increase the dissolution rate of HK by forming micelles. The HK–PLX (1:4) SD exhibited much better dissolution behavior than free HK, and the percentages of the HK–PLX (1:4) SD release at 5 min were about 33.67% ± 0.19% in the AGJ and 46.18% ± 0.26% in the AIJ. Its release percentage reached a maximum of 100% at 45 min in the AGJ and at 30 min in the AIJ. As can be seen from the results, the HK–PLX (1:4) SD was obviously faster than both the PM and the free HK in AGJ and AIJ. The increased dissolution rate of the HK–PLX (1:4) SD was mainly attributed to the fact that the HK existed in the SD in an amorphous state and formed intermolecular hydrogen bonds with PLX. Therefore, the HK–PLX (1:4) SD preparation could be rapidly absorbed after oral administration of HK formulations and produced a better response for its clinical application. 

### 3.4. Pharmacokinetic Behavior of HK Formulations

**Figure 9:**
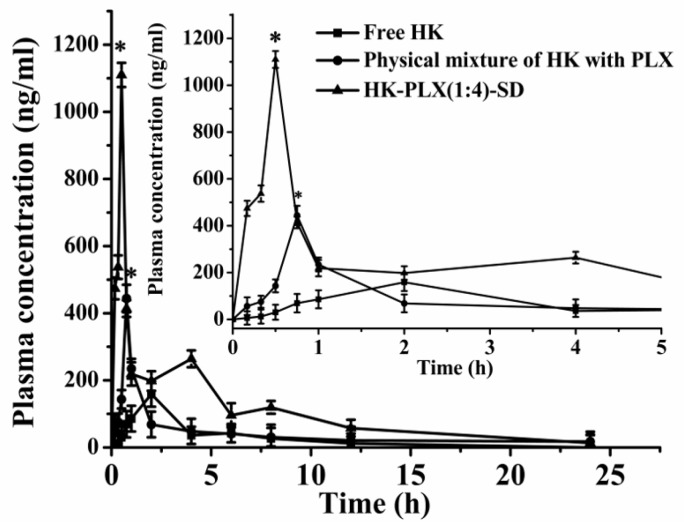
The bioavailability result of the free HK, the physical mixture and the HK–PLX (1:4) SD Data are presented as the mean ± standard deviation (*n* = 6). * *p* < 0.05 vs. free HK.

Plasma concentration–time profiles and corresponding pharmacokinetic parameters of HK, PM of HK with PLX, and the HK–PLX (1:4) SD in rats are displayed in Figure 9 and Table 1, respectively. HK content in the plasma was determined by the HPLC method. As shown in Figure 9, the PM of HK with PLX showed a slightly higher plasma concentration than the free HK, while the HK–PLX (1:4) SD displayed significantly higher plasma concentrations of HK compared to the PM and the free HK. The difference between the groups was statistically significant (*p* < 0.05). Additionally, the *C*_max_ values of the free HK, the PM, and the HK–PLX (1:4) SD were 159.02 ± 5.65 ng/mL, 443.81 ± 9.14 ng/mL, and 1109.87 ± 7.24 ng/mL, respectively. The AUC values of the free HK, the PM, and the HK–PLX (1:4) SD were 580.45 ± 11.15 ng/mL·h, 848.34 ± 12.24 ng/mL·h, and 2558.22 ± 8.15 ng/mL·h, respectively. The results illustrated that the AUC and the *C*_max_ values of the PM were slightly higher than those of the free HK, while the HK–PLX (1:4) SD gave significantly higher AUC and *C*_max_ values compared to the PM and the free HK. Moreover, the value of *C*_max_ for the HK–PLX (1:4) SD was about 6.98-fold greater than that of free HK, and the AUC(0→*t*) of the HK–PLX (1:4) SD was about 4.41-fold greater than that of free HK. Furthermore, the half-life (*t*_1/2_) of the HK–PLX (1:4) SD (0.37 ± 0.05 h) was shorter than that of the PM (0.51 ± 0.12 h) and the free HK (0.74 ± 0.15 h). The *T*_max_ of the HK–PLX (1:4) SD (0.5 ± 0.08 h) was also shorter than that of the PM (0.75 ± 0.09 h) and the free HK (2 ± 0.15 h). Thus, we concluded that the HK–PLX (1:4) SD had good oral bioavailability in rats compared to the PM of HK with PLX and the free HK. 

Furthermore, it had been reported that the honokiol nanosuspensions were prepared by the precipitation–ultrasonication method, and the honokiol existed as an amorphous form in the honokiol nanosuspensions [27]. The pharmacokinetic test results in rats showed that the honokiol nanosuspension had a higher AUC(0→*t*) value in rats, which was about 2.2 times that of HK–CMCS. However, in this study, the in vivo pharmacokinetics in rats demonstrated that the AUC(0→*t*) value of the HK–PLX (1:4) SD preparation was approximately 4.41-fold greater than that of free HK, which indicated that the HK–PLX (1:4) SD should be the potential oral delivery system for clinical applications and could be better absorbed by the body.

### 3.5. MTT Study

**Figure 10:**
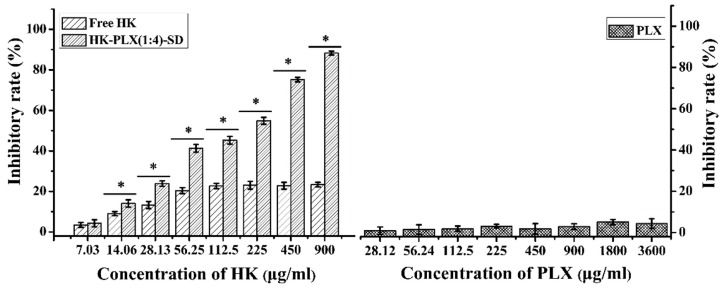
Inhibitory rate of the free HK, the HK–PLX (1:4) SD, and the corresponding PLX (* *p* < 0.05).

In this study, the inhibitory effect of the HK–PLX (1:4) SD on HepG2 cell lines was studied and compared with free HK. Cell viabilities of HepG2 cells after 48 h incubation with different concentrations of the HK–PLX (1:4) SD, free HK, and PLX are shown in Figure 10. It was found that the PLX had little inhibitory effect on the growth of the cell. However, both the HK–PLX (1:4) SD and the free HK have certain inhibitory effects on HepG2 cell growth, and with the increase of drug concentration, the ability to inhibit HepG2 cell growth was significantly improved. When the concentration exceeded 56.25 μg/mL, the inhibitory effect of the free HK on cells did not change significantly, which might be due to the low solubility of free HK. The HK–PLX (1:4) SD showed more effective inhibition than free HK and the difference between the groups was statistically significant (*p* < 0.05), which could be explained by the increased cellular uptake of the HK SD formulation.

## 4. Conclusions

The SD formulation of HK with PLX ((1:4)) was prepared by the solvent evaporation method, and characterized by SEM, FTIR, XRD, and DSC. The SEM analysis illustrated that the SD of HK exhibited different morphology than free HK, and no HK crystals were observed in the SD. The XRD and DSC results illustrated that the HK existed in the SD in amorphous form. The FTIR spectra indicated that there might be intermolecular hydrogen bonds between HK and PLX. Moreover, the solubility and dissolution experiments in AGJ and AIJ clearly indicated that the HK–PLX (1:4) SD had the higher solubility, with values of 32.43 ± 0.36 mg/mL and 34.41 ± 0.38 mg/mL, respectively. The dissolution rate of the HK–PLX (1:4) SD was also apparently higher than free HK in AGJ and AIJ. Moreover, the *C*_max_ value and the AUC(0→*t*) value of the HK–PLX (1:4) SD (1109.87 ± 7.24 ng/mL and 2558.22 ± 8.15 ng/mL·h, respectively) were about 6.98- and 4.41-fold higher than those of free HK (159.02 ± 5.65 ng/mL and 580.45 ± 11.15 ng/mL·h, respectively); hence, the HK–PLX (1:4) SD had better bioavailability than free HK. MTT study showed that the HK–PLX (1:4) SD generated higher inhibition of HepG2 cells than free HK. In addition, the solvent residual of the HK–PLX (1:4) SD was suitable for pharmaceutical use. Thus, it can be concluded that the HK–PLX (1:4) SD prepared in this study significantly improved the absorption of HK in vivo, and has potential clinical application value as a new oral drug formulation.

## Figures and Tables

**Table 1:** Pharmacokinetic parameters for HK in rats after oral administration of free HK, physical mixture of HK with PLX, and HK–PLX (1:4) SD. Data are presented as mean ± standard deviation (*n* = 6). * *p* < 0.05 (vs. free HK).

Pharmacokinetic Parameters	Free HK	Physical Mixture of HK and PLX	HK–PLX (1:4) SD
*C*_max_ (ng/mL)	159.02 ± 5.65	443.81 ± 9.14 *	1109.87 ± 7.24 *
*T*_max_ (h)	2 ± 0.15	0.75 ± 0.09 *	0.5 ± 0.08 *
t1/2 (h)	0.74 ± 0.15	0.51 ± 0.12	0.37 ± 0.05 *
MRT(0–t) (h)	3.93 ± 1.25	6.94 ± 2.22 *	5.70 ± 0.76 *
MRT(0–∞) (h)	5.11 ± 2.13	12.15 ± 3.15 *	6.44 ± 1.15
AUC(0–t) (ng/mL·h)	580.45 ± 11.15	848.34 ± 12.24 *	2558.22 ± 8.15 *
AUC(0–∞) (ng/mL·h)	637.91 ± 13.42	1004.11 ± 15.38 *	2588.70 ± 10.54 *

Maximum plasma concentration(Cmax); Area under the curve (AUC(0–t), AUC(0–∞)); Half time of life (t1/2); Time to peak (Tmax); Mean residence time (MRT(0–t), MRT(0–∞)).

## Supplementary Materials

The following are available online at https://www.mdpi.com/1999-4923/11/11/573/s1, Figure S1: XRD diagram of the SDs of the HK prepared at different time, (a) a week ago, and (b) 16 months ago.
