# Supplementary material for: Highly Water-Soluble Solid Dispersions of Honokiol: Preparation, Solubility, and Bioavailability Studies and Anti-Tumor Activity Evaluation
Source: Pharmaceutics. 2019 Nov 1;11(11):573. doi: 10.3390/pharmaceutics11110573 (PMC6920775; doi:10.3390/pharmaceutics11110573)
Supplement: Supplementary file 1 [file pharmaceutics-11-00573-s001.pdf]

pharmaceutics

Highly Water-Soluble Solid Dispersions of Honokiol: Preparation, Solubility, and Bioavailability Studies and Anti-Tumor Activity Evaluation

Li Wang[1,2], Weiwei Wu[1,2], Lingling Wang[1,2], Lu Wang[1,2] and Xiuhua Zhao[1,2,*]

1. Stability of the HK–PLX (1:4) SD

In order to investigate the stability of the HK–PLX (1:4) SD, the crystal structures of the HK SD prepared in different batches were investigated, as shown in Figure S1. Figure S1a is an XRD diagram of the HK SD prepared one week prior. Figure S1b is an XRD diagram of HK SD prepared 16 months prior. It can be seen from the figure that the crystal structure of the two SDs shows no obvious change, the main characteristic peaks at 2 θ = 19.11° and 23.18° are basically the same as that of PLX, and no other characteristic peaks appear. This shows that the stability of the HK–PLX (1:4) SD was better.

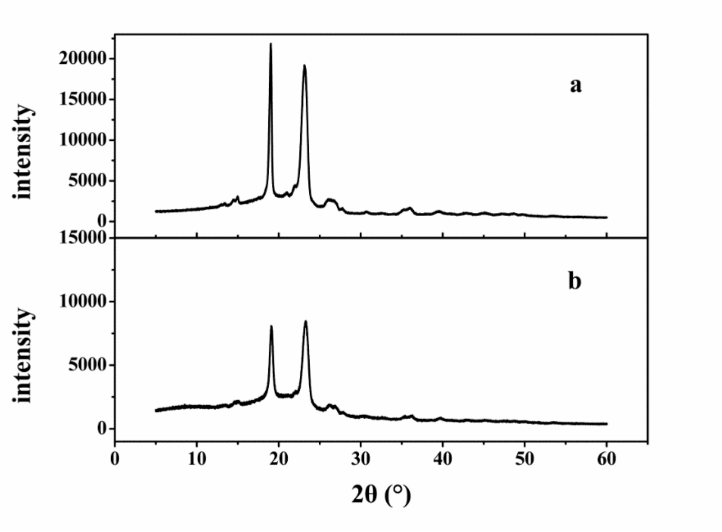

Figure 1. XRD diagram of the HK SD prepared (**a**) one week prior and (**b**) 16 months prior.
